# Laboratory Data Analysis of Hemorrhagic Fever With Renal Syndrome Patients to Predict Disease Severity and Patient Prognosis

**DOI:** 10.1002/jcla.70126

**Published:** 2025-10-27

**Authors:** Hong Shi, Feng Du, Ting Wang, Zhendong Gu, Ting Ruan, Qiujian Zhao, Rui Xu, Yi Wang, Langxi Luo, Shaohua Wang, Liejun Jiang, Yaoni Li

**Affiliations:** ^1^ Department of Laboratory Medicine Baoji Central Hospital Baoji Shaanxi China; ^2^ Department of Infectious Diseases Baoji Central Hospital Baoji Shaanxi China; ^3^ Department of Laboratory Medicine The People's Hospital of Guangxi Zhuang Autonomous Region Nanning Guangxi China

**Keywords:** disease progress, disease severity, hemorrhagic fever with renal syndrome (HFRS), laboratory data, prognosis

## Abstract

**Background:**

Hemorrhagic fever with renal syndrome (HFRS) is an endemic disease occurring in various parts of the world. Prompt access to care with proper treatment is essential for preventing patients from developing renal failure and unfavorite outcomes. This study aimed to elucidate laboratory parameters associated with HFRS severity and prognosis to predict disease course and initiate prompt clinical management.

**Methods:**

Retrospective analysis of laboratory data was performed on HFRS patients from December 2016 to January 2022 in Baoji City of Shaanxi Province in China using different statistical methods.

**Results:**

The WBC and neutrophils in peripheral blood, RBC in urine sediments, blood, protein, and glucose in urine, PT/INR, aPTT, TT, AST, ALT, AST/ALT, MAO, AD, urea, creatinine, cystatin C, CK‐MB, LDH, α‐HBDH, mAST, triglycerides, glucose, amylase, ferritin, and PCT in serum increased in HFRS patients along with disease severity, while the lymphocytes, monocytes, platelets, plateletcrit, fibrinogen, serum total protein, albumin, HDL‐c, magnesium, complement C3 and C4, IgG, triiodothyronine, thyroxine, and free triiodothyronine were reduced. Results also indicated that in uncured HFRS patients, the NEUT%, CRP, cast, PT/INR, aPTT, D‐dimer, AST/ALT, CK‐MB, LDH, α‐HBDH, m‐AST, ferritin, and PCT were significantly higher than in cured patients, while platelets, C3, and C4 in uncured patients were significantly lower than in cured patients. The NEUT%, CRP, AST/ALT, and LDH were associated with patients' prognosis.

**Conclusions:**

Laboratory data are helpful in predicting HFRS patients' progress, severity, and prognosis, thus, these parameters are useful in guiding prompt clinical management of patients.

## Graphical abstract

Prompt access to care with proper treatment is essential for preventing patients from developing renal failure and unfavorable outcomes. This study aimed to elucidate laboratory parameters associated with HFRS severity and prognosis to predict disease course and initiate prompt clinical management. Retrospective analysis of laboratory data was performed on HFRS patients from December 2016 to January 2022 in Baoji City of Shaanxi Province in China using different statistical methods. Multiple abnormal laboratory results were found in patients with HFRS when compared with healthy subjects. The WBC and neutrophils in peripheral blood, RBC in urine sediments, blood, protein, and glucose in urine, PT/INR, aPTT, TT, AST, ALT, AST/ALT, MAO, AD, urea, creatinine, cystatin C, CK‐MB, LDH, α‐HBDH, mAST, triglycerides, glucose, amylase, ferritin, and PCT in serum increased along with disease severity, while the lymphocytes, monocytes, platelets, plateletcrit, fibrinogen, serum total protein, albumin, HDL‐c, magnesium, complement C3 and C4, IgG, triiodothyronine, thyroxine, and free triiodothyronine were reduced. Results also indicated that in uncured HFRS patients, the NEUT%, CRP, cast, PT/INR, aPTT, D‐dimer, AST/ALT, CK‐MB, LDH, α‐HBDH, m‐AST, ferritin, and PCT were significantly higher than in cured patients, while platelets, C3, and C4 in uncured patients were significantly lower than in cured patients. The NEUT%, CRP, AST/ALT, and LDH were associated with patients' prognosis. Laboratory data are helpful in predicting HFRS patients' progress, severity, and prognosis; thus, these parameters are useful in guiding prompt clinical management of patients.

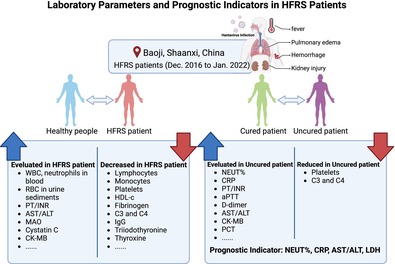

**Abbreviations**
AD: adenosine deaminase
ALT: alanine aminotransferase
aPTT: activated partial thromboplastin time
AST: aspartate transferase
AST/ALT: aspartate transferase and alanine aminotransferase ratio
CK‐MB: creatinine kinase MB
CRP: C‐reactive protein
HDL‐c: high‐density lipoprotein cholesterol
IgG: immunoglobulin G
INR: International Normalized Ratio
LDH: lactate dehydrogenase
MAO: monoamine oxidase
mAST: mitochondrial‐AST
NEUT%: neutrophil percent
PCT: procalcitonin
PT: prothrombin time
PT/INR: prothrombin time International Normalized Ratio
RBC: red blood cell
TT: thrombin time
WBC: white blood cell
α‐HBDH: α‐hydroxybutyrate dehydrogenase

## 1 Introduction

Hemorrhagic fever with renal syndrome (HFRS) caused by Hantavirus infection occurs endemically in many Asian and European countries [1, 2, 3, 4, 5, 6]. Hantaviruses are RNA viruses belonging to the Bunyaviridae family and cause two types of clinical syndrome: hemorrhagic fever with renal syndrome in Asia and Europe and hantavirus cardiopulmonary syndrome in North America, Central America and South America [7]. HFRS affects about 200,000 humans annually worldwide [1, 8], and China alone had 9187 cases in 2021 [9]. The reservoirs of Hantaviruses are rodents. Hantavirus‐induced diseases are emerging zoonoses with endemic appearances and frequent outbreaks in various parts of the world. Humans are infected with Hantavirus by contact with or exposure to the aerosolized urine, feces, or saliva of rodents [10]. The symptoms of hemorrhagic fever with renal syndrome include high fever, hemorrhage, and renal damage; China has the highest number of HFRS cases worldwide and accounts for over 90% of the total reported cases [11]. It is believed that the pathogenesis of HFRS is Hantavirus‐caused acute kidney injury leading to transient proteinuria, which is aroused by the disruption of the glomerular filtration barrier due to vascular leakage caused by the virus. Studies found that in humans, hantaviral infection disrupted the endothelial cell barrier, which was followed by increased capillary permeability, thrombocytopenia due to platelet activation/depletion, and an overactive immune response [12]. However, direct infection of endothelial cells by orthohantaviruses did not increase endothelial permeability [13, 14]. In addition, renal damage from the virus infection could lead to chronic renal damage. Mortality of hemorrhagic fever and HFRS is reduced significantly if patients are aware of the disease and have convenient access to care with prompt and proper clinical management. Thus, prompt diagnosis and assessment of the patient's condition are important for good treatment outcomes and reduction of long‐term sequelae. Here, we retrospectively analyzed the large laboratory data of HFRS patients collected between December 2016 and January 2021 in Baoji City of Shaanxi Province in the northwest of China to find risk factors and elucidate details of the pathogenesis of patients during the disease course.

## 2 Patients and Methods

### 2.1 Patients

HFRS infections occurring between December 2016 and January 2022 in Baoji City were enrolled in this retrospective analysis. A total of 687 cases were enrolled. After exclusion of 3 cases with incomplete laboratory testing, 684 cases were eligible for the statistical analysis. For a better comparison with minimal bias, laboratory data of 473 healthy subjects from corresponding periods were collected for analysis. For patient demographics, 518 cases were male, and 166 cases were female, with ages spanning from 8 to 92 and 5 to 83 years, respectively. For healthy subjects, 259 were male and 178 were female with ages spanning from 25 to 90 and 19 to 85 years, respectively.

### 2.2 Diagnosis of HFRS

Diagnosis of HFRS was made according to a consensus from the Infectious Diseases Prevention and Control Committee of the Association of Chinese Preventive Medicine and the Infectious Diseases Committee of the Chinese Medical Association regarding the diagnosis of Hemorrhagic Fever with Renal Syndrome [15] along with the Ministry of Health of the People's Republic of China Diagnostic Criteria for Epidemic Hemorrhagic Fever (WS 278‐2008) [16].

### 2.3 Ethics/ IRB Approval

This study was approved by the Ethics Committee/Institutional Review Board (IRB) of Baoji Central Hospital (No. BZYL2022‐47) for the collection and analysis of patients' information. The consent agreement from patients was waived because the study was retrospective and data were collected without release of patients' identifications.

### 2.4 Hematology Analysis (Complete Blood Count and Indices Analysis)

Peripheral blood was collected upon admission for hospitalization using the EDTA.K2 blood collection tube (Jiangsu Kangjian Medical Products Co. Ltd., Taizhou, Jiangsu, China). Samples were delivered to the clinical laboratory for analysis within 1 h after collection, and complete blood count and indices analysis were performed on a BC6800Plus and BC7500CS (Mindray Corporation, Shenzhen, Guangdong, China) following the manufacturer's instructions and routine standard operating procedures.

### 2.5 Urine Chemistry and Sedimentation Analysis

The midstream urine was collected, and urine chemistry analysis was performed on an H800 platform (Dirui Industrial Co. Ltd., Changchun, Jilin, China) Urine sedimentation analysis was performed on an UF‐1000i platform (Sysmex Corporation, Kobe, Japan) following the manufacturer's instructions and routine standard operating procedures.

### 2.6 Blood Chemistry Analysis

Fasting venous blood was collected on the day following hospitalization using the blood collection tube from Lingen Precision Medical Products Co. Ltd. (Shanghai, China). Samples were delivered to the clinical laboratory within 1 h after collection. Liver function panel, renal function panel, endocrine panel, infectious diseases immunological panel, iron panel, nutrient panel, cardiac panel, lipid panel, thyroid function panel, and other analytes were analyzed on the Roche Cobas 8000 system (Roche diagnostics USA, Indianapolis, Indiana, USA), the Alinity platform (Abbott Laboratories, Green Oaks, Illinois, USA), and the Cobas E6000 (Roche diagnostics USA) following the manufacturer's instructions and routine standard operating procedures. Briefly, blood samples were centrifuged at 3000 rpm for 10 min at room temperature to separate serum; the samples were then placed on the automatic chemistry analyzer for analysis. Results were verified and reported by a licensed clinical laboratory technologist; data were automatically recorded in the Laboratory Information System for further analysis.

### 2.7 Hemostasis and Coagulation Analysis

Peripheral blood was collected as required using the sodium citrate blood collection tube (Jiangsu Kangjian Medical Products Co. Ltd.). Samples were delivered to the clinical laboratory within 1 h after collection. Samples were prepared as described in 2.6 to separate the plasma and the hemostasis and coagulation analysis (prothrombin time, PT; activated partial thrombin time, aPTT; thrombin time, TT; fibrinogen, FIB; D‐dimer, DD; fibrinogen degradation products, FDP) was then analyzed on a CS 5100 system (Sysmex Corporation, Kobe, Japan) following the manufacturer's instructions and routine standard operating procedures. Data was automatically recorded in the Laboratory Information System for further analysis.

### 2.8 C‐Reactive Protein Analysis

C‐reactive protein (CRP) was analyzed on the Aristo analyzer (Goldsite Diagnostics Inc., Shenzhen, Guangdong, China) using peripheral blood (whole blood) after hematology analysis following the manufacturer's instructions and routine standard operating procedures. Data was automatically recorded in the Laboratory Information System for further analysis.

### 2.9 Flow Cytometry Analysis

Peripheral blood was collected using the EDTA.K2 blood collection tube (Jiangsu Kangjian Medical Products Co. Ltd., Taizhou, Jiangsu, China) and was delivered to the clinical laboratory within 1 h after collection. Flow cytometry analysis for T cell subsets was performed on a BD FACS Canto II flow cytometer (Beckton Dickinson, Franklin Lakes, New Jersey, USA) following the manufacturer's instructions and routine standard operating procedures. Fluorescence dye‐labeled anti‐CD3, CD4, CD8, CD16, CD19, and CD56 antibodies were provided by BD Biosciences. Data were analyzed using the FACSCanto software. Data were automatically recorded in the Laboratory Information System for further analysis.

### 2.10 Serum Protein Electrophoresis

Serum protein electrophoresis was performed using the chemistry samples on a SEBIA Capillarys 3 TERA (SEBIA, Lisses, France) following the manufacturer's instructions and routine standard operating procedures. Briefly, the automatic analysis started by placing serum‐separated blood tubes into the sample loading rack, the barcode was read by the analyzer, and serum was sampled into the capillary, starting the capillary electrophoresis. The electropherogram and data were recorded and saved automatically.

### 2.11 Statistical Analysis

SPSS version 26.0 was used for statistical analysis. The Shapiro–Wilk test was used to evaluate if the samples were normally distributed, and the Independent‐Samples *T* Test or the Mann–Whitney *U* Test was used for analysis accordingly. Mean and Standard Deviation (STDEV), or Mean Rank and Sum of Ranks were compared and presented in all tables for corresponding analysis. All data were analyzed by a third‐party statistical service. A *p* value < 0.05 was considered statistically significant.

## 3 Results

### 3.1 Demographics of Patients and Healthy Subjects

The demographics of HFRS patients and healthy subjects are shown in Table S1.

### 3.2 Comparison of Hematology Analysis Results Between HFRS Patients and Healthy Subjects

Results showed that white blood cells (WBC), monocyte count percentage (MONO%), mean corpuscular hemoglobin (MCH), mean corpuscular hemoglobin concentration (MCHC), red blood cell distribution width (RDW‐CV), and C‐reactive protein (CRP) in HFRS patients were significantly higher than in healthy subjects (*p* < 0.001, < 0.001, < 0.001, < 0.001, 0.022, and < 0.001, respectively), while the lymphocyte count percentage (LYM%), hemoglobin (HGB), red blood cells (RBC), hematocrit (HCT), mean corpuscular volume (MCV), platelets (PLT), plateletcrit (PCT), mean platelet volume (MPV), and platelet distribution width (PDW) in patients were significantly lower than in healthy subjects (*p* < 0.001, for all) (Table 1).

**TABLE 1:** Comparison of hematology parameters between the infection group and healthy control group.

Parameters	Groups	*n*	Mean	STDEV	*t*	*p*
WBC (×10^9^/L)	Infection	684	13.5	9.5	−19.902	< 0.001
	Controls	473	6.1	1.6		
NEUT%	Infection	684	59.9	17.3	−1.071	0.284
	Controls	473	59.1	8.4		
LYM%	Infection	684	23.1	11.7	14.698	< 0.001
	Controls	473	31.5	7.8		
MONO%	Infection	684	12.7	6.8	−22.114	< 0.001
	Controls	473	6.6	2.1		
HGB (g/L)	Infection	684	141.2	24.2	5.543	< 0.001
	Controls	473	147.5	14.6		
RBC (×10^12^/L)	Infection	684	4.5	0.8	8.955	< 0.001
	Controls	473	4.8	0.5		
HCT (%)	Infection	684	40.3	6.7	13.428	< 0.001
	Controls	472	44.4	3.8		
MCV (fL)	Infection	684	89.6	7.0	6.435	< 0.001
	Controls	473	91.9	5.3		
MCH (pg)	Infection	684	31.4	2.2	−6.548	< 0.001
	Controls	473	30.5	2.2		
MCHC (g/L)	Infection	684	350.9	20.1	−20.315	< 0.001
	Controls	473	331.9	11.7		
RDW‐CV (%)	Infection	684	13.3	1.2	−2.299	0.022
	Controls	473	13.1	1.0		
PLT (×10^9^/L)	Infection	684	60.3	53.5	47.743	< 0.001
	Controls	473	221.3	58.3		
PCT (%)	Infection	576	0.1	0.1	45.635	< 0.001
	Controls	466	0.3	0.1		
MPV (fL)	Infection	576	11.1	1.3	14.010	< 0.001
	Controls	472	12.5	1.7		
PDW (%)	Infection	576	15.2	3.4	8.569	< 0.001
	Controls	463	17.0	3.2		
CRP (mg/L)	Infection	669	32.6	31.7	−5.390	< 0.001
	Controls	21	9.9	18.5		

*Note:* The Independent‐Samples *T* Test was applied for this analysis.

Abbreviations: CRP, C‐reactive protein; HCT, hematocrit; HGB, hemoglobin; LYM%, lymphocyte percentage; MCH, mean corpuscular hemoglobin; MCHC, mean corpuscular hemoglobin concentration; MCV, mean corpuscular volume; MONO %, monocyte percentage; MPV, mean platelet volume; NEUT%, neutrophil percentage; PCT, plateletcrit; PDW, platelet distribution width; PLT, platelet; RBC, red blood cell; RDW‐CV, red cell distribution width; STDEV, standard deviation; WBC, white blood cell.

### 3.3 Comparison of Urine Analysis Results Between HFRS Patients and Healthy Subjects

Results indicated that in urine analysis, the casts, epithelial cells, red blood cells, and white blood cells in urine sediments in HFRS patients were significantly elevated compared to healthy subjects (*p* < 0.001, for all); protein, urine occult blood, bilirubin, glucose, ketone, nitrite, and urobilinogen in patients were significantly higher than in healthy subjects (*p* < 0.001, < 0.001, < 0.001, < 0.001, < 0.001, 0.040, and < 0.001, respectively); the urine specific gravity in patients was significantly lower than in healthy subjects (*p* < 0.001). (Table 2).

**TABLE 2:** Comparison of urinalysis parameters between the infection group and healthy control group.

Parameters	Groups	*n*	Mean rank	Sum of ranks	Mann–Whitney *U*	*Z*	*p*
Cast (*n*/HPF)	Infection	676	758.8	512,970.0	34,928.0	−24.563	< 0.001
	Controls	472	310.5	146,556.0			
EC (*n*/μL)	Infection	676	777.0	525,242.0	22,656.0	−26.416	< 0.001
	Controls	472	284.5	134,284.0			
RBC (*n*/μL)	Infection	676	777.0	525,242.0	22,656.0	−26.416	< 0.001
	Controls	472	284.5	134,284.0			
WBC (*n*/μL)	Infection	676	690.4	466,714.0	81,184.0	−17.707	< 0.001
	Controls	472	408.5	192,812.0			
PRO (− to 4+)	Infection	676	775.5	524,244.0	23,654.0	−27.139	< 0.001
	Controls	472	286.6	135,282.0			
BLD (− to 4+)	Infection	676	699.1	472,614.0	75,284.0	−18.287	< 0.001
	Controls	472	396.0	186,912.0			
BIL (− to 4+)	Infection	676	591.1	399,588.5	148,309.5	−5.745	< 0.001
	Controls	472	550.7	259,937.5			
GLU (− to 4+)	Infection	676	595.8	402,758.0	145,140.0	−6.702	< 0.001
	Controls	472	544.0	256,768.0			
KET (− to 4+)	Infection	676	614.4	415,307.0	132,591.0	−9.230	< 0.001
	Controls	472	517.4	244,219.0			
NIT (− to 4+)	Infection	676	577.7	390,518.0	157,380.0	−2.052	0.040
	Controls	472	569.9	269,008.0			
SG	Infection	676	524.1	354,319.5	125,493.5	−6.316	< 0.001
	Controls	472	646.6	305,206.5			
URO (− to 4+)	Infection	676	612.6	414,123.0	133,775.0	−8.995	< 0.001
	Controls	472	519.9	245,403.0			

*Note:* The Mann–Whitney *U* Test was applied for this analysis.

Abbreviations: BIL, bilirubin; BLD, blood; EC, epithelial cell; GLU, glucose; KET, ketone; NIT, nitrites; PRO, protein; SG, specific gravity; URO, urobilinogen.

### 3.4 Comparison of Chemistry Analysis Results Between HFRS Patients and Healthy Subjects

Results showed that serum levels of direct bilirubin, total bile acid, urea, creatinine, uric acid, cystatin C, triglyceride, enzyme activities including α‐l‐fucosidase, alanine transaminase (ALT), aspartate transferase (AST), mitochondrial aspartate transferase (m‐AST), creatine kinase (CK), creatine kinase‐myocardial band (CK‐MB), lactate dehydrogenase (LDH), and α‐hydroxybutyrate dehydrogenase (α‐HBDH) in patients were significantly higher than in healthy subjects (*p* < 0.001, for all), while serum indirect bilirubin, total protein, albumin, globulin, cholinesterase activity, total cholesterol, high‐density lipoprotein cholesterol (HDL‐C), and apolipoprotein A1 and B in patients were significantly lower than in healthy subjects (*p* < 0.001, for all). However, there were no significant differences in the levels of total bilirubin, low‐density lipoprotein cholesterol (LDL‐C), and γ‐glutamyl transferase (γ‐GT) activity between patients and healthy subjects (*p* = 0.823, 0.685, and 0.332, respectively) (Table 3).

**TABLE 3:** Comparison of chemistry parameters between the infection group and healthy control group.

Parameters	Groups	*n*	Mean	STDEV	*t*	*p*
TBIL (μmol/L)	Infection	672	12.5	11.5	−0.224	0.823
	Controls	473	12.6	5.1		
DBIL (μmol/L)	Infection	672	6.5	8.5	5.431	< 0.001
	Controls	473	4.7	1.6		
IBIL (μmol/L)	Infection	672	5.9	4.3	−7.875	< 0.001
	Controls	473	7.9	3.7		
TP (g/L)	Infection	671	57.7	21.7	−19.513	< 0.001
	Controls	365	74.5	4.0		
ALB (g/L)	Infection	672	33.9	5.0	−58.326	< 0.001
	Controls	365	48.1	2.8		
GLO (g/L)	Infection	671	23.2	5.4	−10.780	< 0.001
	Controls	365	26.4	4.0		
ALT (U/L)	Infection	667	64.0	164.7	6.032	< 0.001
	Controls	473	25.3	17.2		
AST (U/L)	Infection	673	121.3	410.2	6.298	< 0.001
	Controls	473	21.7	9.1		
GGT (U/L)	Infection	657	40.5	42.7	0.405	0.685
	Controls	43	37.9	28.4		
TBA (μmol/L)	Infection	670	6.9	9.3	9.039	< 0.001
	Controls	364	3.4	2.7		
CHE (U/L)	Infection	656	5207.3	1504.0	−19.108	< 0.001
	Controls	43	9746.4	1586.2		
AFU (U/L)	Infection	672	26.1	7.4	3.750	< 0.001
	Controls	364	24.5	5.7		
UREA (mmol/L)	Infection	684	14.5	21.7	11.967	< 0.001
	Controls	473	4.6	1.1		
CRE (μmol/L)	Infection	684	235.0	201.1	21.537	< 0.001
	Controls	473	68.7	15.6		
UA (μmol/L)	Infection	537	417.7	202.0	9.786	< 0.001
	Controls	470	323.5	89.1		
Cys‐C (mg/L)	Infection	683	2.8	2.0	19.197	< 0.001
	Controls	8	0.9	0.2		
CK (U/L)	Infection	675	259.7	699.4	4.128	< 1
	Controls	82	125.6	164.8		
CK‐MB (U/L)	Infection	679	28.1	38.8	9.817	< 0.001
	Controls	82	12.5	5.1		
LDH (U/L)	Infection	672	575.9	489.2	21.250	< 0.001
	Controls	81	170.4	25.3		
a‐HBDH (U/L)	Infection	673	391.3	257.6	25.375	< 0.001
	Controls	81	134.4	17.7		
m‐AST (U/L)	Infection	675	40.4	56.4	14.787	< 0.001
	Controls	82	7.7	3.8		
CHO (mmol/L)	Infection	650	2.53	0.88	−36.866	< 0.001
	Controls	457	4.55	0.91		
TG (mmol/L)	Infection	651	2.27	2.37	5.514	< 0.001
	Controls	457	1.69	1.05		
HDL‐C (mmol/L)	Infection	651	0.66	0.27	−39.427	< 0.001
	Controls	457	1.35	0.30		
LDL‐C (mmol/L)	Infection	498	2.46	9.82	−0.991	0.322
	Controls	426	2.89	0.82		
Apo‐A1 (g/L)	Infection	492	0.81	0.95	−11.885	< 0.001
	Controls	294	1.48	0.25		
Apo‐B (g/L)	Infection	492	0.75	0.73	−4.757	< 0.001
	Controls	294	0.96	0.25		

*Note:* The Independent‐Samples *T* Test was applied for this analysis.

Abbreviations: AFU, α‐l‐fucosidase; ALB, albumin; ALT, alanine transaminase; Apo‐A1, apolipoprotein A1; Apo‐B, apolipoprotein B; AST, aspartate transferase; CHE, cholinesterase; CHO, total cholesterol; CK, creatine kinase; CK‐MB, creatine kinase‐myocardial band; CRE, creatinine; Cys‐C, cystatin C; DBIL, direct bilirubin; GGT, γ‐Glutamyl transferase; GLO, globulin; HDL‐C, high‐density lipoprotein cholesterol; IBIL, indirect bilirubin; LDH, lactate dehydrogenase; LDL‐C, low‐density lipoprotein cholesterol; m‐AST, mitochondrial aspartate transferase; TBA, total bile acid; TBIL, total bilirubin; TG, triglyceride; TP, total protein; UA, uric acid; α‐HBDH, α‐hydroxybutyrate dehydrogenase.

### 3.5 Association Between Hematology Analytical Results and Disease Severity

**FIGURE 1:**
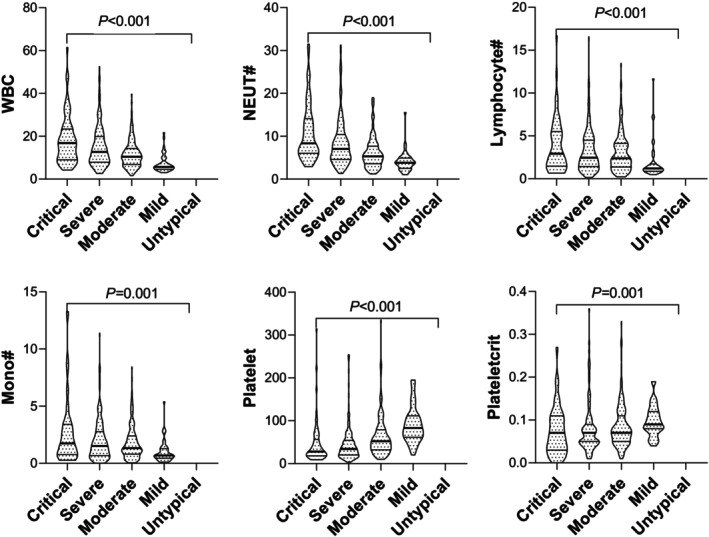
Association between hematology test results and disease severity. Independent‐Sample Kruskal–Wallis Test results showed that the association between WBC, neutrophil counts (NEUT#), lymphocyte counts (LYM#), monocyte counts (MONO#), platelets, and plateletcrit (PCT) and disease severity. WBC and neutrophils increased along with the disease severity (from mild, moderate, severely ill, and critically ill) in HFRS patients, while the lymphocytes, monocytes, platelets, and plateletcrit were reduced.

Results show that WBC and neutrophils increased along with the disease severity (mild, moderate, severely ill, and critically ill) in HFRS patients, while the lymphocytes, monocytes, platelets, and plateletcrit were reduced with disease severity (Figure 1).

### 3.6 Association Between Urine Analytical Results and Disease Severity

**FIGURE 2:**
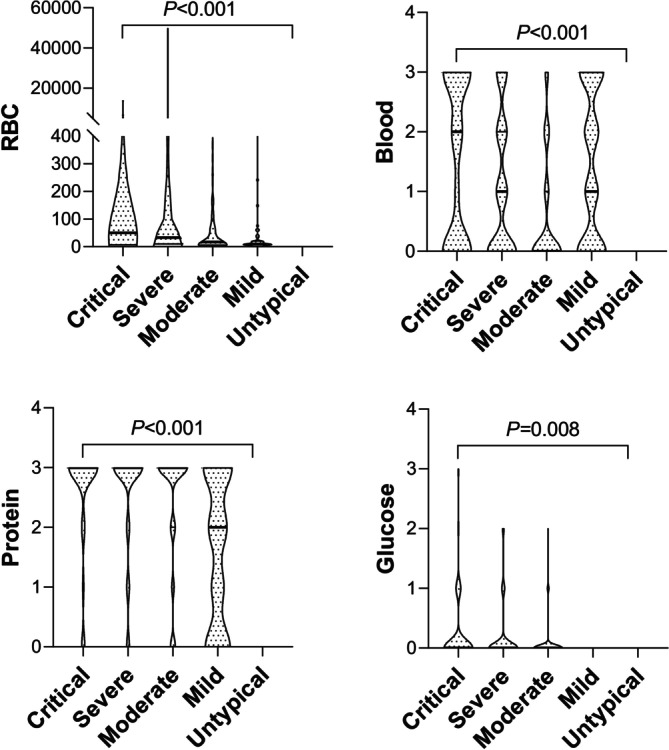
Association between urine analytical results and disease severity. Independent‐Sample Kruskal–Wallis Test results showed that RBC in urine sediments and protein, blood, and glucose in urine chemistry were increased along with the disease severity (from mild, moderate, severely ill, and critically ill) in HFRS patients.

RBC in urine sediments and occult blood, protein, and glucose in urine chemistry were increased along with disease severity in HFRS patients (Figure 2).

### 3.7 Association Between Hemostasis and Coagulation Test Results and Disease Severity

**FIGURE 3:**
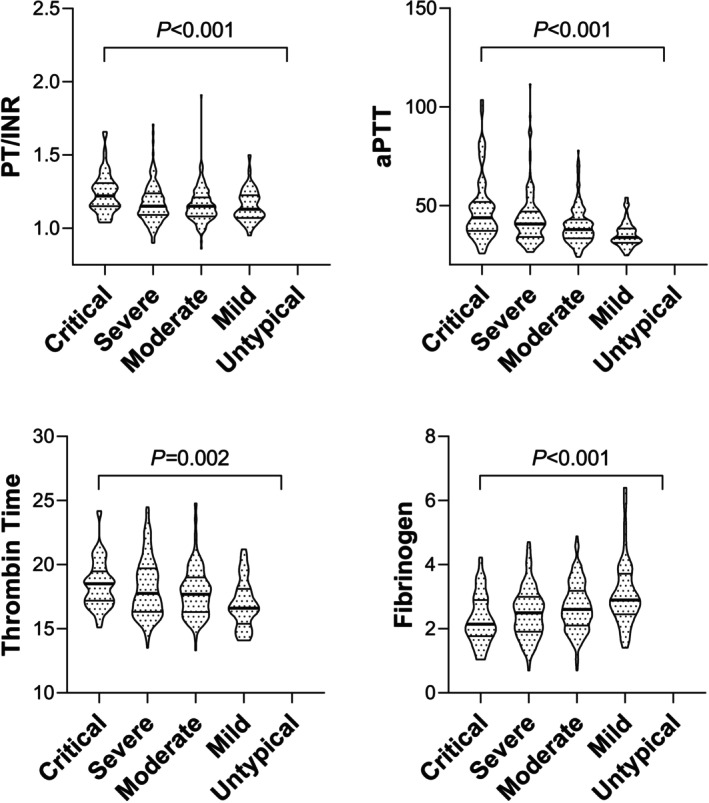
Association between hemostasis and coagulation test results and disease severity. Independent‐Sample Kruskal–Wallis Test results showed that PT‐INR, aPTT, and TT were increased along with the disease severity (from mild, moderate, severely ill, and critically ill) in patients, while the fibrinogen was reduced.

PT‐INR, aPTT, and TT were increased along with the disease severity in patients, while the fibrinogen was reduced (Figure 3).

### 3.8 Association Between Blood Chemistry Analytical Results and Disease Severity

**FIGURE 4:**
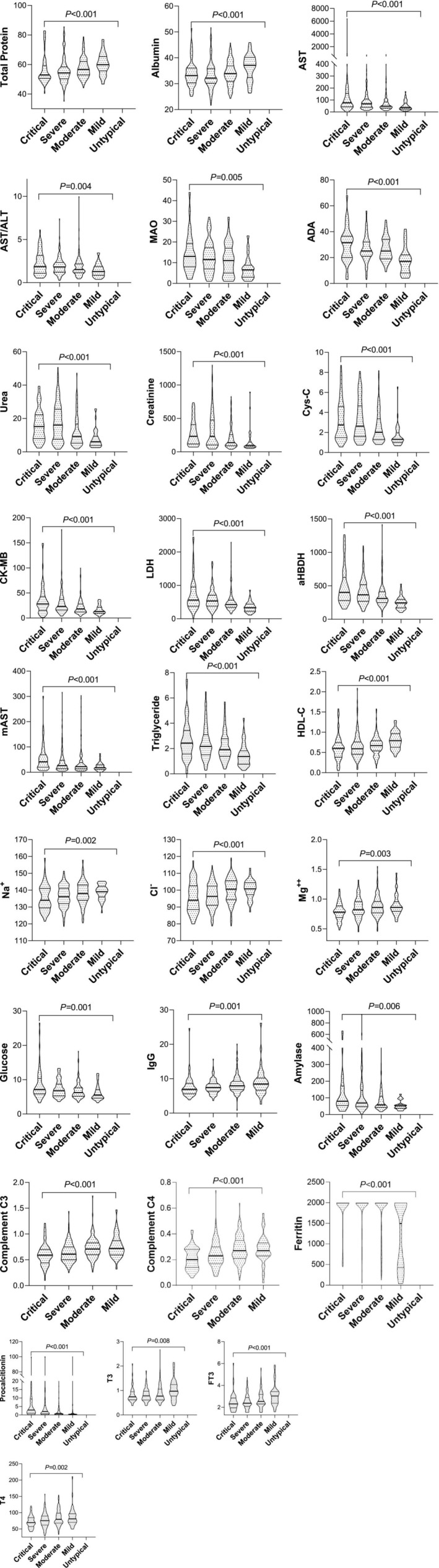
Association between chemistry analytical results and disease severity. Independent‐Sample Kruskal–Wallis Test results showed that total serum protein, albumin, HDL cholesterol, sodium, chloride, magnesium, complement C3 and C4, immunoglobulin G, triiodothyronine, thyroxine, and free triiodothyronine were reduced along with the disease severity (from mild, moderate, severely ill, and critically ill) in patients, while the aspartate transferase (AST), AST/ALT, monoamine oxidase, adenosine deaminase, urea, creatinine, cystatin C, creatinine kinase MB, lactate dehydrogenase, α‐Hydroxybutyrate dehydrogenase, m‐AST, triglycerides, glucose, amylase, ferritin, and procalcitonin were increased. We were able to classify 187 cases with disease course, of which, 27 had fever, 5 with hypotensive shock, 25 with oliguria, 23 with polyuria, 14 with recovery, 93 with mixed stages. 187 cases had hematology, urine sedimentation, coagulation, liver function, kidney function, cardiac enzymes, thyroid hormones, immune function, electrolytes, lipids, and tumor biomarkers analysis. Data was analyzed by using the Independent‐Samples Kruskal–Wallis Test (stepwise step‐down), 57 parameters had statistical significance, of which, 8 with 3 or more subsets, these results are helpful for the evaluation of disease course.

Serum total protein, albumin, HDL‐C, sodium, chloride, magnesium, complement C3 and C4, immunoglobulin G, triiodothyronine, thyroxine, and free triiodothyronine were reduced along with the disease severity in patients, while the AST, AST/ALT, monoamine oxidase (MAO), adenosine deaminase (AD), urea, creatinine, cystatin C, CK‐MB, LDH, α‐HBDH, m‐AST, triglycerides, glucose, amylase, ferritin, and procalcitonin were increased with disease severity (Figure 4).

### 3.9 Association Between Laboratory Parameters and Patients' Outcomes

In uncured HFRS patients, the NEUT%, CRP, cast, PT‐INR, aPTT, D‐dimer, AST/ALT, CK‐MB, LDH, α‐HBDH, m‐AST, ferritin, and PCT were significantly higher than in cured patients (*p* = 0.002, 0.002, 0.036, 0.001, 0.001, 0.001, 0.003, 0.001, < 0.001, < 0.001, 0.001, < 0.001, and 0.006, respectively), while PLT and Complement C3 and C4 in uncured patients were significantly lower than in cured patients (*p* = 0.054, 0.001, and 0.002, respectively) (Table S2).

### 3.10 Patient Outcome Predicting Model

**FIGURE 5:**
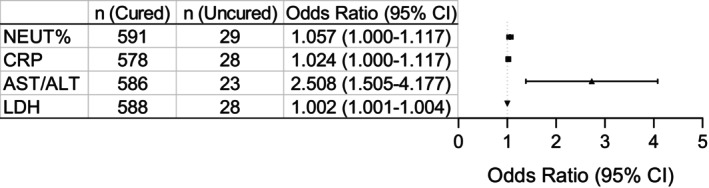
Binary Logistic Regression analysis for patients' prognosis. Six‐hundred and twenty (620) HFRS patients with treatment outcomes, of which 591 cases were cured or improved, 29 cases were uncured or died. Data was analyzed with univariate analysis first, then multivariate analysis was performed when the difference was significant. These variates were NEUT%, MONO%, PLT, CRP, CAST, PT/INR, aPTT, D‐Dimer, AST/ALT, CK‐MB, LDH, a‐HBDH, m‐AST, complements C3 and C4, ferritin, and PCT. Among these parameters, NEUT%, CRP, AST/ALT, and LDH were independent predictors for patient's prognosis. Model formula: −12.609 + 0.055*NEUT% + 0.024*CRP + 0.919*AST/ALT + 0.002*LDH.

All laboratory parameters were analyzed, and results showed that with 108 laboratory parameters, 64 parameters were associated with patients' severity, and 60 parameters were associated with clinical course. Of these, 47 parameters were associated with both disease severity and clinical course (Table S3). Multivariate analysis showed that only NEUT%, CRP, AST/ALT, and LDH were associated with patients' prognosis, and all the Odd Ratios (OS) were higher than 1 (Figure 5). The model formula was: −12.609 + 0.055*NEUT% + 0.024*CRP + 0.919*AST/ALT + 0.002*LDH.

## 4 Discussion

The mortality rate of HFRS has decreased in recent years due to improving medical and healthcare services and public awareness in the endemic area. Prompt diagnosis and management are critical in maintaining a low death rate, and laboratory parameters are important indicators in guiding diagnosis and treatment. The following are the findings of this investigation.

Our study results indicated that hematology analysis was an important tool in predicting the severity of HFRS. Increased WBC and CRP indicated an infection status due to possible bacterial co‐infection. Increased MONO% may be attributed to viral infection of monocytes, which behaved as virus reservoirs and induced production of inflammatory cytokines [17, 18]. The elevation of MCH, MCHC, and RDW‐CV suggested a status of anemia of various origins [19, 20, 21, 22, 23, 24, 25, 26]. A decrease in HGB, RBC, HCT, and MCV further suggested an anemic state in patients [25, 27, 28]. The low PLT, PCT (plateletcrit), MPV, and PDW in patients may have been indicators of disease severity (survival or death) [29, 30]; however, there was no report on the relationship between low PLT, PCT, MPV, and PDW and HFRS. One report mentioned that MPV, immature platelet fraction (IPF%), prothrombin fragments, and D‐dimer together with serum thrombopoietin (TPO) were elevated in acute HFRS patients with active thrombopoiesis, but these parameters did not predict the severity of HFRS [31]. Further study is needed to interpret this phenomenon.

Increased urine sediment elements—the casts, epithelial cells, red blood cells, and white blood cells; chemistry parameters protein, blood, bilirubin, glucose, ketone, nitrite, and urobilinogen; and low urine specific gravity in HFRS patients reflected the possible damage to renal function [32, 33, 34, 35] and the malfunction of metabolisms such as liver function [36, 37].

Elevation of serum levels of multi‐parameters—direct bilirubin, total bile acid, urea, creatinine, uric acid, cystatin C, triglyceride, enzyme activities including α‐l‐fucosidase, alanine transaminase, aspartate transferase, mitochondrial aspartate transferase, creatine kinase, creatine kinase‐myocardial band, lactate dehydrogenase, and α‐hydroxybutyrate dehydrogenase, and reduction of serum indirect bilirubin, total protein, albumin, globulin, cholinesterase activity, total cholesterol, high‐density lipoprotein cholesterol, and apolipoprotein A1 and B in HFRS patients further indicated the multi‐organ damage and malfunction of metabolism [36, 37].

The association between WBC, neutrophils in peripheral blood, RBC in urine sediments and blood, protein, glucose in urine chemistry increment, reduction of lymphocytes, monocytes, platelets, and plateletcrit and HFRS severity could be valuable in predicting HFRS severity and guiding proper management.

Similarly, PT‐INR, aPTT, and TT were increased along with disease severity in patients, while the fibrinogen was reduced. This could be explained by Hantavirus attacking microvasculature in multiple organs, including the endothelial cells of blood vessels, causing malfunction of thrombosis as well as many parameters in serum chemistry analysis [36] (Figure 4).

In addition, in the attempt to correlate the disease course with laboratory parameters, we found that subsets in a total of 187 cases had aberrant laboratory parameters, which were associated with the disease course. These further suggested the importance of laboratory parameters in the prediction of patients' severity and outcome.

Lastly, only a few parameters, the NEUT%, CRP, AST/ALT, and LDH were associated with patients' prognosis (Figure 5).

## 5 Conclusions

Multiple laboratory parameters in hematology analysis, hemostasis and coagulation testing, urine analysis, and chemistry analysis were associated with HFRS patients' progress, severity, and prognosis; thus, they may be helpful in evaluating patients' conditions and guiding prompt and proper clinical management for increased treatment efficacy.

## 6 Limitations of This Study

Since this was a retrospective study that analyzed HFRS cases that occurred between December 2016 and January 2022 in Baoji City of Shaanxi Province in China, many factors influenced the completeness of laboratory testing results; thus, many patients' laboratory data were incomplete and ineligible for analysis. We were unable to compare laboratory parameters between pre‐ and post‐ treatment for all patients. Many laboratory parameters were incomplete for some patients either because of treatment abandonment or the fact that patients did not have relevant clinical evidence to receive the tests. Some test results were unable to be analyzed because of the lack of associated test results from healthy controls (not required for the health check‐up). We were unable to follow up with the patients after hospital discharge for long‐term renal function and recovery evaluations.

## Author Contributions

Yaoni Li, Feng Du, Ting Wang, Zhendong Gu, Ting Ruan, Rui Xu, Hong Shi, Yi Wang, Liangxi Luo, and Shaohua Wang were involved in the investigation and data collection; Yaoni Li and Qiujian Zhao were project organizers; Liejun Jiang performed the statistical analysis; and Huayi Huang conceptualized the study and wrote the manuscript.

## Ethics Statement

This study was approved by the Institutional Review Board/Ethics Committee of Baoji Central Hospital for collecting healthcare data (Hospital No. BZYL2022‐47). Informed consent was not required for this study.

## Consent

The authors have nothing to report.

## Conflicts of Interest

The authors declare no conflicts of interest.

## Supporting information


**Data S1:** jcla70126‐sup‐0001‐Supinfo01.docx.

## Data Availability

The data that support the findings of this study are available from the corresponding author upon reasonable request due to privacy or ethical restrictions.
