# Supplementary material for: Laboratory Data Analysis of Hemorrhagic Fever With Renal Syndrome Patients to Predict Disease Severity and Patient Prognosis
Source: J Clin Lab Anal. 2025 Oct 27;39(23):e70126. doi: 10.1002/jcla.70126 (PMC12699194; doi:10.1002/jcla.70126)
Supplement: Supplementary file 1 — Data S1: jcla70126‐sup‐0001‐Supinfo01.docx. [file JCLA-39-e70126-s001.docx]

**Table S1 Average age of infection and healthy control groups**

| Objects | Sex | n | Age range (years) | Mean (years) |
| --- | --- | --- | --- | --- |
| Infection group |  | 684 |  |  |
|  | M | 518 | 8-92 | 51.5 |
|  | F | 166 | 5-83 | 60.5 |
| Healthy control group |  | 473 |  |  |
|  | M | 295 | 25-90 | 47.0 |
|  | F | 178 | 19-85 | 46.0 |

| **Table S2** **Association between laboratory parameters and patient’s outcome**   \| Parameters \| Prognosis \| n \| Mean \| STDEV \| *t* \| *P* \| \| --- \| --- \| --- \| --- \| --- \| --- \| --- \| \| NEUT% \| Uncured \| 29 \| 68.9 \| 14.2 \| 3.051 \| 0.002 \| \|  \| Cured \| 591 \| 59.1 \| 16.9 \|  \|  \| \| PLT (×10^9^/L) \| Uncured \| 29 \| 40.1 \| 39.3 \| -1.933 \| 0.054 \| \|  \| Cured \| 591 \| 58.6 \| 50.8 \|  \|  \| \| CRP (mg/L) \| Uncured \| 28 \| 55.9 \| 37.8 \| 3.448 \| 0.002 \| \|  \| Cured \| 578 \| 31.0 \| 28.3 \|  \|  \| \| Cast (n/LPF) \| Uncured \| 25 \| 4.9 \| 7.7 \| 2.222 \| 0.036 \| \|  \| Cured \| 590 \| 1.5 \| 5.3 \|  \|  \| \| PT-INR \| Uncured \| 29 \| 1.5 \| 0.5 \| 3.779 \| 0.001 \| \|  \| Cured \| 587 \| 1.2 \| 0.4 \|  \|  \| \| aPTT (second) \| Uncured \| 29 \| 58.8 \| 25.2 \| 3.547 \| 0.001 \| \|  \| Cured \| 587 \| 42.1 \| 14.8 \|  \|  \| \| D-Dimer (mg/L) \| Uncured \| 28 \| 10.2 \| 9.9 \| 3.563 \| 0.001 \| \|  \| Cured \| 536 \| 3.5 \| 5.7 \|  \|  \| \| AST/ALT \| Uncured \| 23 \| 3.6 \| 2.4 \| 3.327 \| 0.003 \| \|  \| Cured \| 586 \| 1.9 \| 1.3 \|  \|  \| \| CK-MB (U/L) \| Uncured \| 29 \| 91.2 \| 94.6 \| 3.785 \| 0.001 \| \|  \| Cured \| 588 \| 24.6 \| 18.7 \|  \|  \| \| LDH (U/L) \| Uncured \| 28 \| 1645.8 \| 1264.9 \| 4.682 \| < 0.001 \| \|  \| Cured \| 584 \| 525.1 \| 305.0 \|  \|  \| \| a-HBDH (U/L) \| Uncured \| 28 \| 892.1 \| 587.1 \| 4.719 \| < 0.001 \| \|  \| Cured \| 583 \| 367.1 \| 189.4 \|  \|  \| \| m-AST (U/L) \| Uncured \| 29 \| 111.0 \| 111.2 \| 3.588 \| 0.001 \| \|  \| Cured \| 585 \| 36.6 \| 41.7 \|  \|  \| \| C3 (mg/dL) \| Uncured \| 23 \| 0.5 \| 0.3 \| -3.401 \| 0.001 \| \|  \| Cured \| 546 \| 0.7 \| 0.2 \|  \|  \| \| C4 (mg/dL) \| Uncured \| 23 \| 0.2 \| 0.1 \| -3.096 \| 0.002 \| \|  \| Cured \| 546 \| 0.3 \| 0.1 \|  \|  \| \| Ferritin (µg/L) \| Uncured \| 20 \| 2000.0 \| 0.0 \| 9.498 \| < 0.001 \| \|  \| Cured \| 533 \| 1705.9 \| 714.9 \|  \|  \| \| PCT (ng/mL) \| Uncured \| 28 \| 19.6 \| 24.8 \| 2.967 \| 0.006 \| \|  \| Cured \| 590 \| 5.6 \| 14.6 \|  \|  \|   The Independent-Samples T Test was used for this analysis. Note: PT-INR, prothrombin time-international normalized; aPTT, activated partial thromboplastin time; C3, complement 3; C4, complement 4; n/LPF: number per low power field  **Table S3 Association between laboratory parameters and the severity and the clinical course of patients** | | | | | | | |
| --- | --- | --- | --- | --- | --- | --- | --- | --- | --- | --- | --- | --- | --- | --- | --- | --- | --- | --- | --- | --- | --- | --- | --- | --- | --- | --- | --- | --- | --- | --- | --- | --- | --- | --- | --- | --- | --- | --- | --- | --- | --- | --- | --- | --- | --- | --- | --- | --- | --- | --- | --- | --- | --- | --- | --- | --- | --- | --- | --- | --- | --- | --- | --- | --- | --- | --- | --- | --- | --- | --- | --- | --- | --- | --- | --- | --- | --- | --- | --- | --- | --- | --- | --- | --- | --- | --- | --- | --- | --- | --- | --- | --- | --- | --- | --- | --- | --- | --- | --- | --- | --- | --- | --- | --- | --- | --- | --- | --- | --- | --- | --- | --- | --- | --- | --- | --- | --- | --- | --- | --- | --- | --- | --- | --- | --- | --- | --- | --- | --- | --- | --- | --- | --- | --- | --- | --- | --- | --- | --- | --- | --- | --- | --- | --- | --- | --- | --- | --- | --- | --- | --- | --- | --- | --- | --- | --- | --- | --- | --- | --- | --- | --- | --- | --- | --- | --- | --- | --- | --- | --- | --- | --- | --- | --- | --- | --- | --- | --- | --- | --- | --- | --- | --- | --- | --- | --- | --- | --- | --- | --- | --- | --- | --- | --- | --- | --- | --- | --- | --- | --- | --- | --- | --- | --- | --- | --- | --- | --- | --- | --- | --- | --- | --- | --- | --- | --- | --- | --- | --- | --- | --- | --- | --- | --- | --- | --- | --- | --- | --- | --- | --- | --- | --- | --- | --- | --- | --- | --- |
|  | Parameters | Disease severity | | | Clinical course | | |
|  |  | n | *H* | *P* | n | *H* | *P* |
| 1 | WBC (×10^9^/L) | 438 | 59.270 | < 0.001 | 187 | 28.665 | < 0.001 |
| 2 | NEUT# (×10^9^/L) | 438 | 72.732 | < 0.001 | 187 | 34.277 | < 0.001 |
| 3 | NEUT% | 438 | 12.251 | 0.016 | 187 | 11.535 | 0.042 |
| 4 | LYM# (×10^9^/L) | 438 | 22.046 | < 0.001 | 187 | 13.628 | 0.018 |
| 5 | LYM% | 438 | 14.190 | 0.007 | 187 | 15.168 | 0.010 |
| 6 | MONO# (×10^9^/L) | 438 | 19.933 | 0.001 | 187 | 18.517 | 0.002 |
| 7 | MONO% | 438 | 5.330 | 0.255 | 187 | 8.395 | 0.136 |
| 8 | EOS# (×10^9^/L) | 438 | 12.318 | 0.015 | 187 | 6.865 | 0.231 |
| 9 | EOS% | 438 | 2.293 | 0.682 | 187 | 5.323 | 0.378 |
| 10 | BASO# (×10^9^/L) | 438 | 21.986 | < 0.001 | 187 | 17.882 | 0.003 |
| 11 | BASO% | 438 | 9.115 | 0.058 | 187 | 11.624 | 0.040 |
| 12 | HGB (g/L) | 438 | 10.903 | 0.028 | 187 | 23.201 | < 0.001 |
| 13 | RBC (×10^12^/L) | 438 | 6.484 | 0.166 | 187 | 18.105 | 0.003 |
| 14 | HCT (%) | 438 | 6.141 | 0.189 | 187 | 19.839 | 0.001 |
| 15 | MCV (fL) | 438 | 7.736 | 0.102 | 187 | 2.158 | 0.827 |
| 16 | MCH (pg) | 438 | 5.685 | 0.224 | 187 | 6.870 | 0.230 |
| 17 | MCHC (g/L) | 438 | 25.235 | < 0.001 | 187 | 9.729 | 0.083 |
| 18 | RDW-CV (%) | 438 | 4.029 | 0.402 | 187 | 3.741 | 0.587 |
| 19 | PLT (×10^9^/L) | 438 | 69.053 | < 0.001 | 187 | 36.856 | < 0.001 |
| 20 | PCT (ng/mL) | 365 | 18.947 | 0.001 | 187 | 26.823 | < 0.001 |
| 21 | MPV (fL) | 365 | 6.647 | 0.156 | 187 | 4.014 | 0.547 |
| 22 | PDW (%) | 365 | 3.037 | 0.552 | 187 | 3.348 | 0.646 |
| 23 | CRP (mg/L) | 432 | 10.051 | 0.040 | 187 | 8.280 | 0.141 |
| 24 | Cast (n/LPF) | 436 | 2.336 | 0.674 | 183 | 6.328 | 0.276 |
| 25 | EC (n/µL) | 436 | 5.332 | 0.255 | 183 | 19.936 | 0.001 |
| 26 | RBC (- to 4+) | 436 | 18.070 | 0.001 | 183 | 22.765 | < 0.001 |
| 27 | WBC (- to 4+) | 436 | 1.921 | 0.750 | 183 | 7.276 | 0.201 |
| 28 | PRO (- to 4+) | 436 | 35.756 | < 0.001 | 183 | 25.556 | < 0.001 |
| 29 | BLD (- to 4+) | 436 | 22.183 | < 0.001 | 183 | 20.525 | 0.001 |
| 30 | BIL (- to 4+) | 436 | 1.322 | 0.858 | 183 | 5.168 | 0.396 |
| 31 | GLU (- to 4+) | 436 | 13.864 | 0.008 | 183 | 5.978 | 0.308 |
| 32 | KET (- to 4+) | 436 | 4.107 | 0.392 | 183 | 5.512 | 0.357 |
| 33 | NIT (- to 4+) | 436 | 6.450 | 0.168 | 183 | 2.360 | 0.797 |
| 34 | pH | 436 | 7.609 | 0.107 | 183 | 2.570 | 0.766 |
| 35 | SG | 436 | 9.517 | 0.049 | 183 | 14.466 | 0.013 |
| 36 | URO (- to 4+) | 436 | 16.106 | 0.003 | 183 | 8.603 | 0.126 |
| 37 | Vit. C (- to 4+) | 436 | 2.040 | 0.728 | 183 | 8.098 | 0.151 |
| 38 | PT-sec (second) | 437 | 20.760 | < 0.001 | 185 | 20.871 | 0.001 |
| 39 | PT (%) | 437 | 19.846 | 0.001 | 185 | 19.616 | 0.001 |
| 40 | PT-INR | 437 | 21.666 | < 0.001 | 185 | 16.306 | 0.006 |
| 41 | aPTT (second) | 437 | 35.226 | < 0.001 | 185 | 21.031 | 0.001 |
| 42 | TT (second) | 437 | 16.976 | 0.002 | 185 | 22.849 | < 0.001 |
| 43 | FIB (g/L) | 437 | 23.708 | < 0.001 | 185 | 31.021 | < 0.001 |
| 44 | D-Dimer (mg/L) | 403 | 32.886 | < 0.001 | 162 | 21.606 | 0.001 |
| 45 | TBIL (µmol/L) | 436 | 14.339 | 0.006 | 181 | 9.240 | 0.100 |
| 46 | DBIL (µmol/L) | 436 | 22.111 | < 0.001 | 181 | 11.236 | 0.047 |
| 47 | IBIL (µmol/L) | 436 | 5.540 | 0.236 | 181 | 5.891 | 0.317 |
| 48 | TP (g/L) | 436 | 24.597 | < 0.001 | 181 | 12.778 | 0.026 |
| 49 | ALB (g/L) | 436 | 20.931 | < 0.001 | 181 | 19.636 | 0.001 |
| 50 | GLU (g/L) | 436 | 9.420 | 0.051 | 181 | 16.202 | 0.006 |
| 51 | ALB/GLO | 436 | 9.032 | 0.061 | 181 | 22.343 | < 0.001 |
| 52 | ALT (U/L) | 435 | 9.336 | 0.053 | 181 | 5.508 | 0.357 |
| 53 | AST (U/L) | 435 | 27.393 | < 0.001 | 181 | 20.554 | 0.001 |
| 54 | AST/ALT | 435 | 15.427 | 0.004 | 181 | 16.301 | 0.006 |
| 55 | ALP (U/L) | 346 | 3.656 | 0.455 | 181 | 4.273 | 0.511 |
| 56 | GGT (U/L) | 430 | 11.698 | 0.020 | 181 | 5.226 | 0.389 |
| 57 | TBA (µmol/L) | 434 | 2.423 | 0.658 | 181 | 5.058 | 0.409 |
| 58 | ADA (U/L) | 241 | 21.316 | < 0.001 | 181 | 4.727 | 0.450 |
| 59 | CHE (U/L) | 428 | 5.822 | 0.213 | 181 | 14.563 | 0.012 |
| 60 | MAO (U/L) | 223 | 14.689 | 0.005 | 181 | 10.183 | 0.070 |
| 61 | AFU (U/L) | 436 | 6.989 | 0.136 | 181 | 0.628 | 0.987 |
| 62 | Urea (mmol/L) | 438 | 52.543 | < 0.001 | 187 | 35.410 | < 0.001 |
| 63 | CRE (µmol/L) | 438 | 41.384 | < 0.001 | 187 | 28.077 | < 0.001 |
| 64 | CO2cp (mmol/L) | 438 | 11.352 | 0.023 | 187 | 15.372 | 0.009 |
| 65 | UA (µmol/L) | 354 | 13.174 | 0.010 | 149 | 6.827 | 0.234 |
| 66 | Cys-C (mg/L) | 437 | 42.229 | < 0.001 | 187 | 37.749 | < 0.001 |
| 67 | CK (U/L) | 436 | 19.325 | 0.001 | 186 | 29.475 | < 0.001 |
| 68 | CK-MB (U/L) | 436 | 34.979 | < 0.001 | 186 | 24.130 | < 0.001 |
| 69 | LDH (U/L) | 433 | 46.472 | < 0.001 | 186 | 36.679 | < 0.001 |
| 70 | a-HBDH (U/L) | 436 | 50.251 | < 0.001 | 184 | 32.583 | < 0.001 |
| 71 | m-AST (U/L) | 436 | 27.310 | < 0.001 | 182 | 15.423 | 0.009 |
| 72 | CHO (mmol/L) | 423 | 6.476 | 0.166 | 173 | 13.065 | 0.023 |
| 73 | TG (mmol/L) | 423 | 30.094 | < 0.001 | 173 | 12.142 | 0.033 |
| 74 | HDL-C (mmol/L) | 423 | 26.919 | < 0.001 | 173 | 21.923 | 0.001 |
| 75 | LDL-C (mmol/L) | 328 | 2.167 | 0.705 | 126 | 16.454 | 0.006 |
| 76 | Apo-A1 (g/L) | 323 | 5.050 | 0.282 | 128 | 3.828 | 0.574 |
| 77 | Apo-B (g/L) | 323 | 1.698 | 0.791 | 128 | 12.473 | 0.029 |
| 78 | K^+^ (mmol/L) | 438 | 4.806 | 0.299 | 187 | 6.336 | 0.275 |
| 79 | Na^+^ (mmol/L) | 438 | 17.357 | 0.002 | 187 | 3.483 | 0.626 |
| 80 | Cl^-^ (mmol/L) | 438 | 30.574 | < 0.001 | 187 | 5.048 | 0.410 |
| 81 | Ca^++^ (mmol/L) | 438 | 11.495 | 0.022 | 187 | 7.775 | 0.169 |
| 82 | Mg^++^ (mmol/L) | 438 | 15.851 | 0.003 | 187 | 8.419 | 0.135 |
| 83 | GLU (mmol/L) | 436 | 19.259 | 0.001 | 186 | 14.549 | 0.012 |
| 84 | AMY (U/L) | 316 | 14.413 | 0.006 | 119 | 9.641 | 0.086 |
| 85 | ASO (IU/mL) | 258 | 6.561 | 0.161 | 170 | 35.966 | < 0.001 |
| 86 | RF (IU/mL) | 242 | 12.206 | 0.016 | 170 | 35.107 | < 0.001 |
| 87 | C3 (mg/dL) | 403 | 32.420 | < 0.001 | 170 | 18.014 | 0.003 |
| 88 | C4 (mg/dL) | 403 | 29.078 | < 0.001 | 170 | 19.358 | 0.002 |
| 89 | IgA (mg/dL) | 403 | 0.712 | 0.870 | 170 | 10.257 | 0.068 |
| 90 | IgG (mg/dL) | 403 | 15.580 | 0.001 | 117 | 13.306 | 0.021 |
| 91 | IgM (mg/dL) | 403 | 5.081 | 0.166 | 132 | 7.730 | 0.172 |
| 92 | AFP (µg/L) | 252 | 5.342 | 0.254 | 103 | 10.069 | 0.073 |
| 93 | CEA (µg/L) | 249 | 5.894 | 0.207 | 132 | 7.730 | 0.172 |
| 94 | CA19-9 (U/mL) | 132 | 7.390 | 0.060 | 103 | 10.069 | 0.073 |
| 95 | Ferritin (ng/mL) | 405 | 37.814 | < 0.001 | 158 | 27.928 | < 0.001 |
| 96 | CD16+CD56 (%) | 88 | 1.951 | 0.583 | 62 | 6.961 | 0.138 |
| 97 | CD19 (%) | 64 | 5.098 | 0.165 | 46 | 5.008 | 0.286 |
| 98 | CD3 (%) | 113 | 1.373 | 0.712 | 78 | 6.113 | 0.295 |
| 99 | CD4 (%) | 113 | 7.863 | 0.049 | 78 | 4.669 | 0.458 |
| 100 | CD8 (%) | 113 | 3.091 | 0.378 | 78 | 5.832 | 0.323 |
| 101 | CD4/CD8 (%) | 113 | 6.183 | 0.103 | 78 | 5.655 | 0.341 |
| 102 | PCT (ng/mL) | 438 | 55.919 | < 0.001 | 185 | 31.032 | < 0.001 |
| 103 | T3 (nmol/L) | 406 | 13.705 | 0.008 | 164 | 16.550 | 0.005 |
| 104 | T4 (nmol/L) | 406 | 17.375 | 0.002 | 164 | 13.444 | 0.020 |
| 105 | FT3 (pmol/L) | 406 | 21.613 | < 0.001 | 164 | 8.990 | 0.109 |
| 106 | FT4 (pmol/L) | 406 | 2.748 | 0.314 | 164 | 7.638 | 0.177 |
| 107 | TSH (µIU/mL) | 406 | 0.890 | 0.926 | 164 | 4.582 | 0.469 |
| 108 | NT-proBNP (pg/mL) | 131 | 7.160 | 0.067 | 49 | 6.619 | 0.251 |

The Independent-Samples Kruskal-Wallis Test was used for this analysis. Note: EOS#, eosinophil number; EOS%, eosinophil percentage; BASO#, basophil number; BASO%, basophil percentage; PT-sec, prothrombin time (seconds); FIB, fibrinogen; ALP, alkaline phosphatase; ADA, adenosine deaminase; MAO, monoamine oxidase; CO2cp, CO2 combining power; AMY, amylase; ASO, anti-streptolysin O antibodies; RF, rheumatoid factor; AFP, alpha-fetoprotein; CEA, carcinoembryonic antigen; CA19-9, carbohydrate antigen 19-9; T3, triiodothyronine; T4, thyroxine; FT3, free triiodothyronine; FT4, free thyroxine; TSH, thyroid-stimulating hormone; NT-proBNP, N-terminal pro B-type natriuretic peptide.
